# Chalcogen Bonds Involving Selenium in Protein Structures

**DOI:** 10.1021/acschembio.1c00441

**Published:** 2021-09-03

**Authors:** Oliviero Carugo, Giuseppe Resnati, Pierangelo Metrangolo

**Affiliations:** † Department of Chemistry, University of Pavia, 27100 Pavia, Italy; ‡ Department of Chemistry, Materials, and Chemical Engineering “Giulio Natta”, Politecnico di Milano, Via L. Mancinelli 7, 20131 Milano, Italy

## Abstract

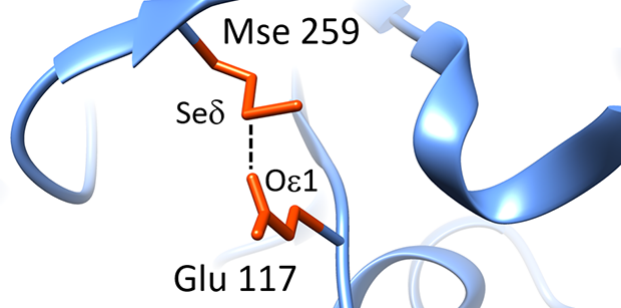

Chalcogen bonds are the specific interactions involving group 16 elements as electrophilic sites. The role of chalcogen atoms as sticky sites in biomolecules is underappreciated, and the few available studies have mostly focused on S. Here, we carried out a statistical analysis over 3562 protein structures in the Protein Data Bank (PDB) containing 18 266 selenomethionines and found that Se···O chalcogen bonds are commonplace. These findings may help the future design of functional peptides and contribute to understanding the role of Se in nature.

The Protein Data Bank (PDB) is a 50-year old worldwide repository of 3D structures of biological macromolecules, including proteins and nucleic acids.^1^ It has nowadays reached over 175 000 structures of proteins, DNA, RNA, and their complexes, which often include metal ions and small molecules (totaling >1 billion atoms). As such, the PDB offers the unique opportunity to analyze protein structures, deriving meaningful statistics about the occurrence of chemical interactions. Protein–ligand and protein–protein binding, as well as interactions governing protein folding, can be analyzed, contributing, e.g., to an understanding of the role of chemical interactions, and potentially unveil yet unknown contributions.^2^ Alongside conventional interactions controlling protein folding, i.e., hydrogen bonding, electrostatic forces, and hydrophobic interactions, more recently, important additional contributions from less common interactions have been identified, including halogen bonding,^3^ chalcogen bonding,^4^ and interactions involving aromatic rings,^5^ e.g., π···π stacking in amyloids,^6^ among others. A full understanding of the role of these noncanonical interactions in protein folding may have important implications, such as in advanced drug design, enhanced drug-target protein affinity,^7^ force fields for improved molecular simulations,^8^ and new insights in biomolecular recognition processes.^9^ The focus of this Letter is on one of these noncanonical interactions, specifically the impact of a chalcogen bond involving selenium (Se) atoms on protein structures.

The term chalcogen bond (ChB) is used to designate the specific subset of inter- and intramolecular interactions formed by chalcogen atoms wherein the group 16 element is the electrophilic site (Figure 1).^10^ Although the earliest examples of chalcogen-bonded complexes can be traced back to 1843,^11^ recently, there has been an upsurge in the number of papers reporting successful examples of the use of this interaction in several fields, such as crystal engineering^12^ and organic catalysis,^13^ among others.^14^ As far as the implications of chalcogen bonding in the biomolecular field are concerned, only in the early 2000s were ChBs found to play a role in protein structures.^15,16^ Sulfur–oxygen ChBs have also been found to occur between the sulfur cation of *S*-adenosylmethionine and oxygen atoms in methyltransferase active sites mediating recognition and catalysis.^17^ Furthermore, some of us have recently demonstrated the importance of this interaction in the inhibition mechanism of maltase glucoamylase by salacinol and katalanol inhibitors, through the ChB between the sulfonium ion center and the catalytic nucleophile Asp443 residue.^18^ It is also known that the biologically important reaction of ebselen, a glutathione peroxidase mimic, with biological cysteine thiol groups is favored by the ability of selenium to act as a ChB donor.^19^ Finally, the PDB has recently been surveyed in order to systematically explore the stabilizing potential of chalcogen bonding in protein–ligand complexes, indicating that chalcogen bonding does indeed play a dominant role in stabilizing some of the interaction motifs studied.^20^ This is relevant in drug design; in fact, a recent survey has demonstrated that this interaction is often isosteric with analogous hydrogen bonding.^7^

**Figure 1:**
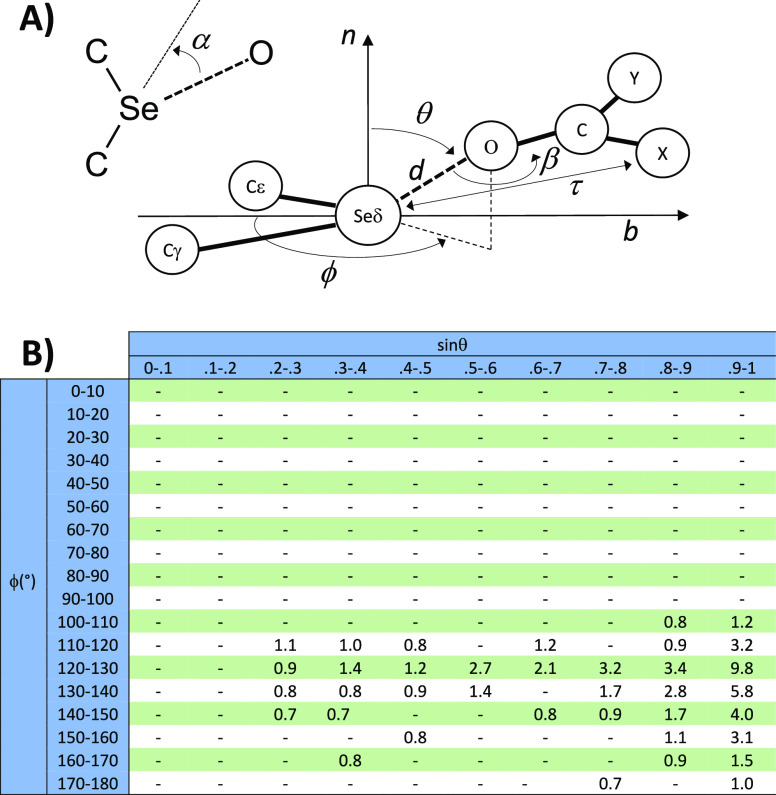
(A) Spherical coordinates (φ, θ, and d) and α angle that describe the position of the oxygen atom relative to the selenium atom and variables (angle β and torsion τ, atoms X–C–O–Se) that describe the orientation of the carbon–oxygen double bonds, acting as ChB acceptors, relative to the selenium atom. (B) Bidimensional distribution of the oxygen atoms around the selenium atom (for clarity, the percentage of observations is shown only for sectors where it is ≥0.6%; see text for details).

ChB analyses in protein structures have mostly focused on sulfur as the donor atom,^21^ either on the protein^22^ or on the ligand.^23^ Se atoms have only occasionally been considered, and, to the best of our knowledge, only from the ligand point of view.^20^ Selenium is seldom present in native proteins,^24^ where it is shown to impart permanent oxidation resistance.^25^ However, Se-containing proteins are well represented in the PDB (nearly 10 000 structures), because incorporation of selenomethionine instead of natural methionine by genetic engineering methods is aiding protein structure phasing, thanks to the anomalous diffraction signal provided by Se.^26^ This gives the unique opportunity to statistically analyze Se-based ChBs that stabilize proteins, since it can be foreseen that Se is a better ChB donor than S, due to its higher polarizability.

With this hypothesis in mind, we extracted from the PDB (March 2021) an ensemble of protein 3D structures containing selenomethionines with the following, commonly accepted, selection criteria:^27^ (*i*) only X-ray crystal structures refined at a resolution better than 2 Å; (*ii*) only structures determined in the 90–110 K temperature range; (*iii*) sequence redundancy was reduced to 40% pairwise sequence identity with CD-HIT.^28^ This resulted in a nonredundant, high-quality, and homogeneous ensemble of 3562 protein chains containing 18 266 selenomethionines.

In the ChBs observed in this ensemble, by far the most represented nucleophilic atoms are oxygen atoms either in the protein backbone or in the amino acid side-chains. Only a few other nucleophilic atoms are present, unless post-translational modifications occur. An oxygen atom was considered to be in contact with the selenium atom if 2 ≤ Se···O distance ≤ 3.5 Å (sum of van der Waals radii = 3.4 Å). A total of 1173 contacts were observed. The position of the oxygen atom relative to the selenium atom has been described through the spherical coordinates shown in Figure 1, similarly to previous studies.^29,30^ The azimuthal angle φ between the bisector of the Cγ–Seδ−Cε angle and the vector from Seδ and the projection of the O atom on the plane defined by Cγ, Seδ, and Cε was constrained in the 0–180° range because of the local, intrinsic *C*_2*v*_ symmetry of the Cγ–Seδ−Cε moiety. For the same reason, the polar angle θ between the normal to the plane defined by Cγ, Seδ, and Cε and the Seδ−O vector was constrained in the 0–90° range. The position of the oxygen atom relative to the selenium atom was also monitored by the angle α, defined as the supplementary of the angle C–Se–O (180°, C–Se–O). This angle provides a piece of very direct information; i.e., the formation of a chalcogen bond is expected to be associated with small α values, close to 0°.

When the O atom approaching the Se atom belonged to a C=O group, the data were divided into five groups. The first with the amido group of the Asn side chains (O = Oδ1, C = Cγ, X = Nδ2, Y = Cβ); the second with the amido group of the Gln side chain (O = Oε1, C = Cδ, X = Nε2, Y = Cγ); the third with the carboxylic group of the Glu side chain (O = Oε1, C = Cδ, X = Oε2, Y = Cγ or, alternatively, O = Oε2, C = Cδ, X = Oε1, Y = Cγ); the fourth with the carboxylic group of the Asp side-chain (O = Oδ1, C = Cγ, X = Oδ2, Y = Cβ or, alternatively, O = Oδ2, C = Cγ, X = Oδ1, Y = Cβ); and the fifth and last one with any backbone carbonyl (O = backbone O, X = backbone Cα, and Y = backbone N of the next residue).

The region around the selenium atom was divided into 180 sectors by dividing the φ range into 18 intervals of 10° each (from 0° to 180°) and by dividing the θ range into 10 equal intervals (from sin θ = 0 to sin θ = 1). In this way, each sector has the same surface, and 0.6% of the oxygen atoms should be observed in each sector if their distribution is continuous and uniform.

Figure 1B clearly shows that this is not the case (for clarity, the percentage of observations is shown only for sectors where it is ≥0.6%). A large fraction of the oxygen atoms is at θ close to 90° and at φ close to 130°. In other words, they approach the selenium atom along a direction opposite that of the carbon atoms bound to the selenium atom, as it is typical for ChBs (Figure 1 and Figures 2A,B). Similar (though not perfectly identical) trends are observed when considering the different types of oxygen atoms, i.e., amide oxygen atoms of Asn and Gln side chains, backbone carbonyl oxygen atoms, carboxylate oxygen atoms of Asp and Glu side chains, and hydroxyl oxygen atoms of Ser, Thr, and Tyr side chains (Supporting Information Figure S1). This observation is confirmed by the relationship between φ and θ shown in Figure 2A, where a clear data clustering appears at θ ≈ 90° and φ ≈ 130°, and data are roughly distributed along an arched curve from low θ and φ values to the center of the aforementioned cluster. In this case, too, no spectacular differences appear from the particular analysis of the different types of oxygen atoms (Supporting Information Figure S2).

**Figure 2:**
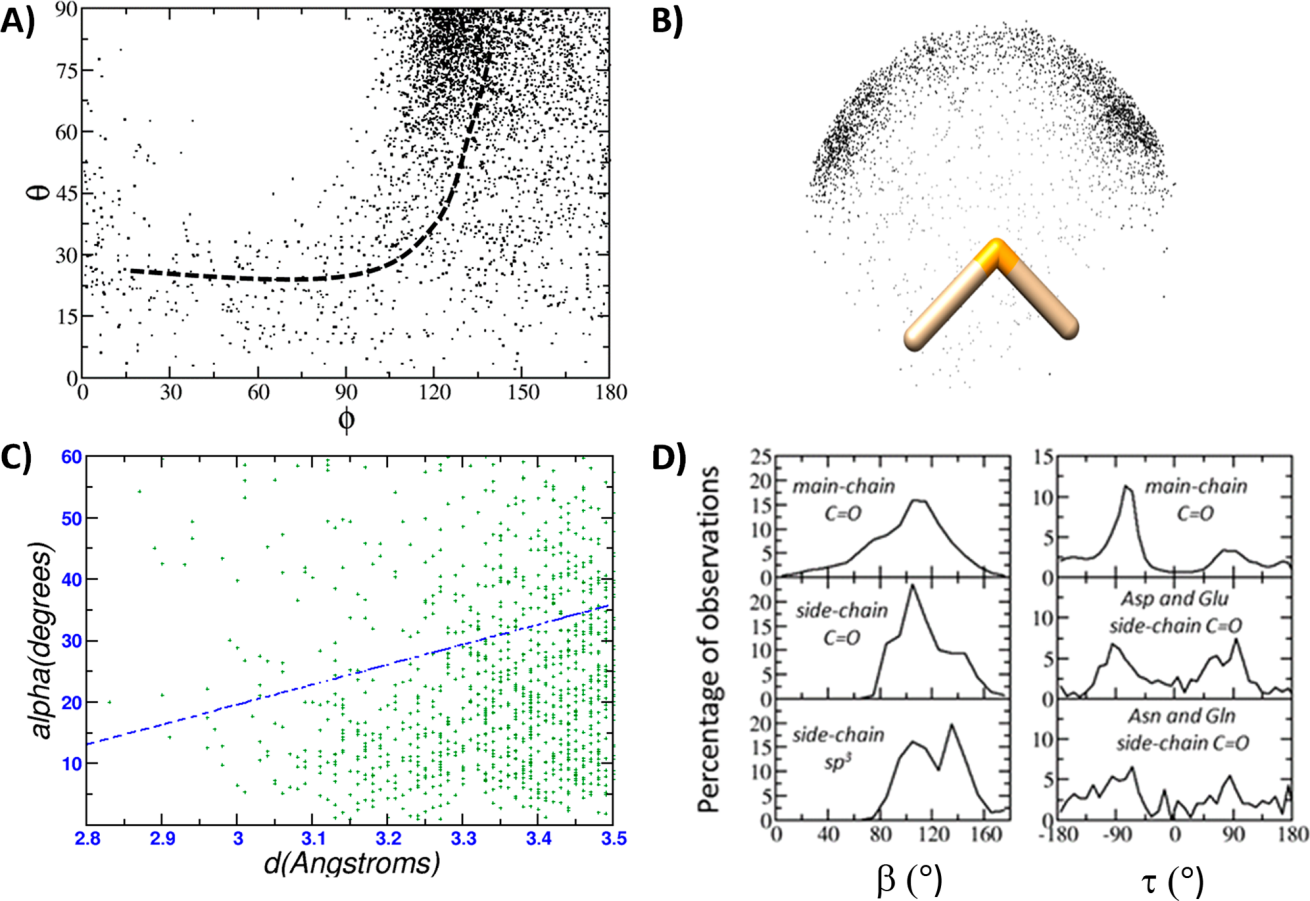
(A) Relationship between the angles θ and φ (deg). (B) Superposition of all the oxygen atoms close to the selenium atom. (C) Relationship between angle α and distance *d* (see definitions in Figure 1A). The broken line indicates the linear relationship (*y* = −62.8 + 29.2*x*; both slope and intercept are significantly different from zero; Pearson correlation coefficient = 0.215). (D) Distribution of the angle β and torsion τ that describe the position of the oxygen around the selenium atom for various types of oxygen atoms.

The superposition of all the oxygen atoms on the same C–Se–C framework shows a clear clustering of the oxygen atoms in the regions opposite that of the C–Se bonds (Figure 2B), and also in this case there are no substantial differences among different types of oxygen atoms (Supporting Information Figure S3). This suggests that the observed selenium–oxygen contacts are, indeed, influenced by chalcogen bonds.

This is supported by the analysis of the angle α values. Given that chalcogen bonds are directional, it is expected that selenium–oxygen contacts are frequently characterized by small α angles and that shorter contacts are associated by smaller α angles. This is observed indeed in the data set of structural data examined here. A total of 43% of the α values are smaller than 20°, and though the α versus *d* scatterplot is quite dispersed (Figure 2C), there is a statistically significant linear relationship between α and *d*. This confirms that chalcogen bonds may be formed by the selenium atom of selenomethionines in proteins.

A total of 65% of the oxygen atoms in contact with the selenium atom are backbone oxygen atoms. A total of 12% are carboxylic oxygen atoms from side chains of Asp and Glu. A total of 8% are amide oxygen atoms from the side chains of Asn and Gln, and the remaining 15% are hydroxylic oxygen atoms from the side chains of Ser, Thr, and Tyr. These values are not very different from the oxygen atom compositions in the proteins examined here: A total of 67% of their oxygen atoms are backbone oxygen atoms. A total of 18% are carboxylic oxygen atoms from side chains of Asp and Glu. A total of 5% are amide oxygen atoms from the side chains of Asn and Gln, and the remaining 10% are hydroxylic oxygen atoms from the side chains of Ser, Thr, and Tyr. The only difference is that carboxylate oxygen atoms are found less frequently than expected in the vicinity of the selenium atoms (12% versus 18%). This is not surprising since the side chains of Asp and Glu are usually solvent-exposed while methionines are usually buried in the protein core. The depletion of charged, carboxylate oxygen atoms is compensated by a modest increase in the percentage of amide and hydroxylic oxygen atoms.

When a chalcogen bond is detected (α ≤ 20°), it is more often opposed to the Se–Cε bond (54%) than to the Se–Cγ bond (46%). This might imply a different electronic density in the two directions. However, it might be a simple consequence of steric hindrance: While the Cε carbon atom is terminal, the Cγ one is connected to the rest of the selenomethionine side chain.

A comprehensive analysis of the geometric features of the studied ChBs requires a detailed analysis of the angular distribution of selenium atoms around oxygen. If this is a tetrahedral sp^3^ hybridized oxygen, like in the hydroxyl groups of Ser, Thr, and Tyr, it is necessary to consider the angle C–O–Se (referred thereinafter to as β). If, on the contrary, it is a sp^2^ hybridized oxygen, like in the carboxylate or amide side chains of Asp, Glu, Asn, and Gln or like in all backbone carbonyls, it is necessary to consider not only the angle β but also the torsion C–C–O–Se (referred thereinafter τ). The distributions of the β and τ values are shown in Figure 2D. The β value distributions are very similar for all types of oxygen atoms, with a broad maximum at about 100–140°. The τ value distributions are similar for all types of oxygen atoms too, but, contrary to the β value distributions that are unimodal, they are bimodal with two maxima, one at about −90° and the other at about +90° (Figure 2D).

Three relevant examples of selenium–oxygen contacts, all due to structural genomics consortia, are shown in Figure 3 and discussed in detail herein. The top of Figure 3 reports the crystal structure of fructokinase from *Xylella fastidiosa* (ID code: 3LKI, chain A).^31^ The selenomethionine 324 interacts with the side-chain oxygen atom of serine 328, and it can be hypothesized that this interaction, which involves two solvent accessible residues that are just five positions apart along the protein sequence, stabilizes the N-terminal moiety of the helix. Another selenium–oxygen contact is observed in the crystal structure of aminopeptidase N from *Neisseria meningitides* (ID code: 2GTQ, chain A).^32^ This interaction involves two residues that are buried in the protein core and are very distant along the protein sequence (seleniomethione 259 and glutamate 117). The third example is observed in the crystal structure of a putative racemase from *Pseudovibrio sp. JE062* (ID code: 3MKC, chain A).^33^ Two residues close in the protein sequence, i.e., selenomethionine 104 and glycine 98, are involved in the interaction, which might stabilize the helix, and, contrary to the previous two examples, involves a backbone oxygen atom instead of a side-chain oxygen atom.

**Figure 3:**
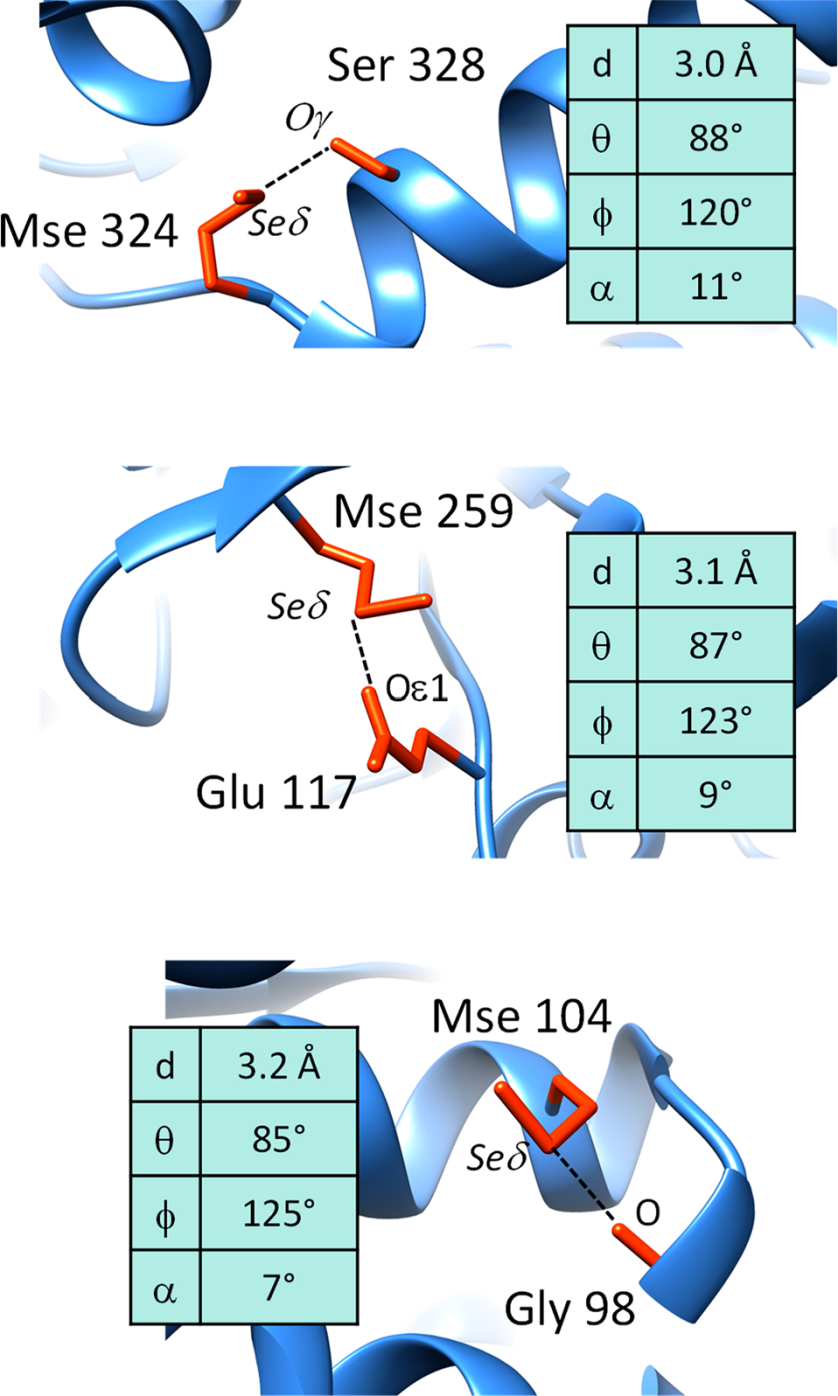
Examples of chalcogen bonds that involve selenomethionine residues in proteins: top, bond between the selenium atom of Mse 324 and the side-chain oxygen atom of Ser 328 (both belonging to chain A of PDB file 3LKI); middle, bond between the selenium atom of Mse 259 and a side-chain oxygen atom of Glu 117 (both belonging to chain A of PDB file 2GTQ); bottom, bond between the selenium atom of Mse 104 and a main-chain oxygen atom of Gly 98 (both belonging to chain A of PDB file 3MKC).

Interestingly, we also found a Se···O ChB in the CSD deposited structure of a heptapeptide, where it was demonstrated that the 3_10_- to α-helical transition is sequence dependent, occurring at O(2) for the peptide incorporating selenomethionine, and shifted by a residue to O(3) for peptides having methionine and S-benzyl cysteine at the same residue.^34^ The observed ChB is within the geometrical parameters observed in Figure 3, i.e., a Se···O distance of 3.351 Å and C–Se···O angle of 150.50° (Figure 4). Importantly, the aforementioned ChB was not recognized by the authors of the original article, and neither the methionine derivative nor the S-benzyl cysteine one display similar interactions involving the chalcogen atoms, which is understandable given the lower polarizability of S with respect to Se.

**Figure 4:**
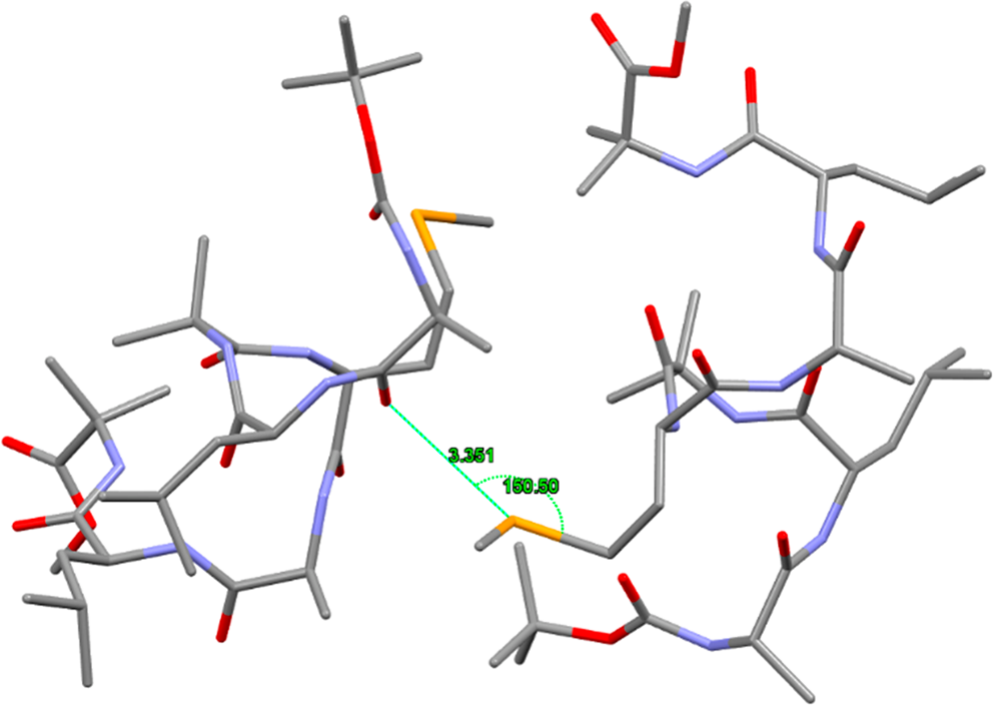
CSD deposited crystal structure of the heptapeptide BocAla-Leu-Aib-Mse-Ala-Leu-Aib-OMe showing the occurrence on an intermolecular Se···O ChB.

In conclusion, by a statistical analysis on a set of PDB crystal structures of proteins containing selenomethionines, we have demonstrated that Se···O chalcogen bonds are commonplace. The interactions explored in this work must be true also for methionine-containing proteins since a SeMet residue does not occur naturally in the structures examined in the PDB, but SeMet is, instead, a replacement for Met in heavy atom replacement experiments to solve structures. However, since the Se atom is more polarizable, one might expect the noncovalent O···Se interactions to be stronger than the corresponding O···S interaction. The results reported in this Letter are relevant for a 2-fold reason. On one hand, this might help provide insights into the chemical basis of selenium-over-sulfur discrimination in nature.^35^ On the other hand, Se···O ChBs can be exploited to manipulate structures and functions of peptides and proteins in synthetic biology. In this respect, Se-functionalized self-assembling peptides are under current investigation.
