# Supplementary material for: Chalcogen Bonds Involving Selenium in Protein Structures
Source: ACS Chem Biol. 2021 Sep 3;16(9):1622–7. doi: 10.1021/acschembio.1c00441 (PMC8453483; doi:10.1021/acschembio.1c00441)
Supplement: Supplementary file 1 — cb1c00441_si_001.pdf [file cb1c00441_si_001.pdf]

Chalcogen Bonds involving Selenium in Protein Structures

Oliviero Carugo,† Giuseppe Resnati,‡ and Pierangelo Metrangolo‡

† Department of Chemistry, University of Pavia, 27100 Pavia, Italy.

‡ Department of Chemistry, Materials, and Chemical Engineering "Giulio Natta", Politecnico di Milano, Via L. Mancinelli 7, 20131 Milano, Italy.

AUTHOR INFORMATION

Corresponding Authors

*Tel.: +39 0382987858. E-mail: olivieroitalo.carugo@unipv.it.

*Tel.: +39 0223993032. E-mail: giuseppe.resnati@polimi.it.

*Tel.: +39 0223993041. E-mail: pierangelo.metrangolo@polimi.it.

SUPPORTING INFORMATION

(a)

ϕ(°)	sinθ									
	0.0-0.1	0.1-0.2	0.2-0.3	0.3-0.4	0.4-0.5	0.5-0.6	0.6-0.7	0.7-0.8	0.8-0.9	0.9-1.0
0-10						3.1		3.1		3.1
10-20										
20-30										
30-40										
40-50										
50-60										
60-70										
70-80										
80-90										
90-100									3.1	
100-110								3.1		
110-120							3.1			
120-130					1.1	6.2	3.1		3.1	6.2
130-140							3.1			12.5
140-150						3.1				
150-160				1.1	1.1	3.1				6.2
160-170				1.1					3.1	6.2
170-180							3.1			3.1

(b)

ϕ(°)	sinθ									
	0.0-0.1	0.1-0.2	0.2-0.3	0.3-0.4	0.4-0.5	0.5-0.6	0.6-0.7	0.7-0.8	0.8-0.9	0.9-1.0
0-10										
10-20										
20-30										
30-40										
40-50										
50-60										
60-70										
70-80										
80-90										
90-100										
100-110										1.4
110-120			1.4	1.6	0.9		1.6		1.4	3.4
120-130			1.6	2.1	1.8	2.8	2.3	3.4	3.9	12.4
130-140				1.1	1.4	0.9	1.1	1.6	3.4	5.3
140-150				0.9				0.9	1.6	4.4
150-160									1.1	3.0
160-170						1.1				1.1
170-180								1.1		2.1

(c)

ϕ(°)	sinθ									
	0.0-0.1	0.1-0.2	0.2-0.3	0.3-0.4	0.4-0.5	0.5-0.6	0.6-0.7	0.7-0.8	0.8-0.9	0.9-1.0
0-10									1.6	
10-20										
20-30										1.6
30-40										
40-50										
50-60										
60-70										
70-80										
80-90										1.8
90-100										1.8
100-110										
110-120				1.8		1.8				5.3
120-130						1.8	1.8	5.3	3.5	1.8
130-140						3.5	1.8	3.5	1.8	8.8
140-150				1.8	1.8	1.8			1.8	1.8
150-160				1.8				1.8	3.5	
160-170									1.8	
170-180							1.8			1.8

(d)

ϕ(°)	sinθ									
	0.0-0.1	0.1-0.2	0.2-0.3	0.3-0.4	0.4-0.5	0.5-0.6	0.6-0.7	0.7-0.8	0.8-0.9	0.9-1.0
0-10						0.8				
10-20										
20-30			0.8							
30-40										
40-50			0.8							
50-60										
60-70					0.7					
70-80										
80-90									0.8	0.8
90-100										0.8
100-110			0.8			0.8		0.8	2.3	1.6
110-120			0.8	0.8	0.8		1.6	0.8		2.3
120-130				2.3	1.6	2.3	1.6	2.3	1.6	5.4
130-140					0.8		1.6	1.6	1.6	4.7
140-150					1.6		3.1	1.6	2.3	4.7
150-160					0.8	1.6	0.8	0.8		3.9
160-170			0.8	0.7	0.8				0.8	2.3
170-180			0.8	0.8				0.8	0.8	1.6

Figure S1. Bidimensional distribution of the oxygen atoms around the selenium atom (for clarity, the percentage of observations is shown only for sectors where it is $\geq$ 0.6%; see main text for details) for amide oxygen atoms of Asn and Gln side-chains (**a**), for backbone carbonyl oxygen atoms (**b**), for carboxylate oxygen atoms of Asp and Glu side-chains (**c**), and for hydroxyl oxygen atoms of Ser, Thr and Tyr side-chains (**d**).

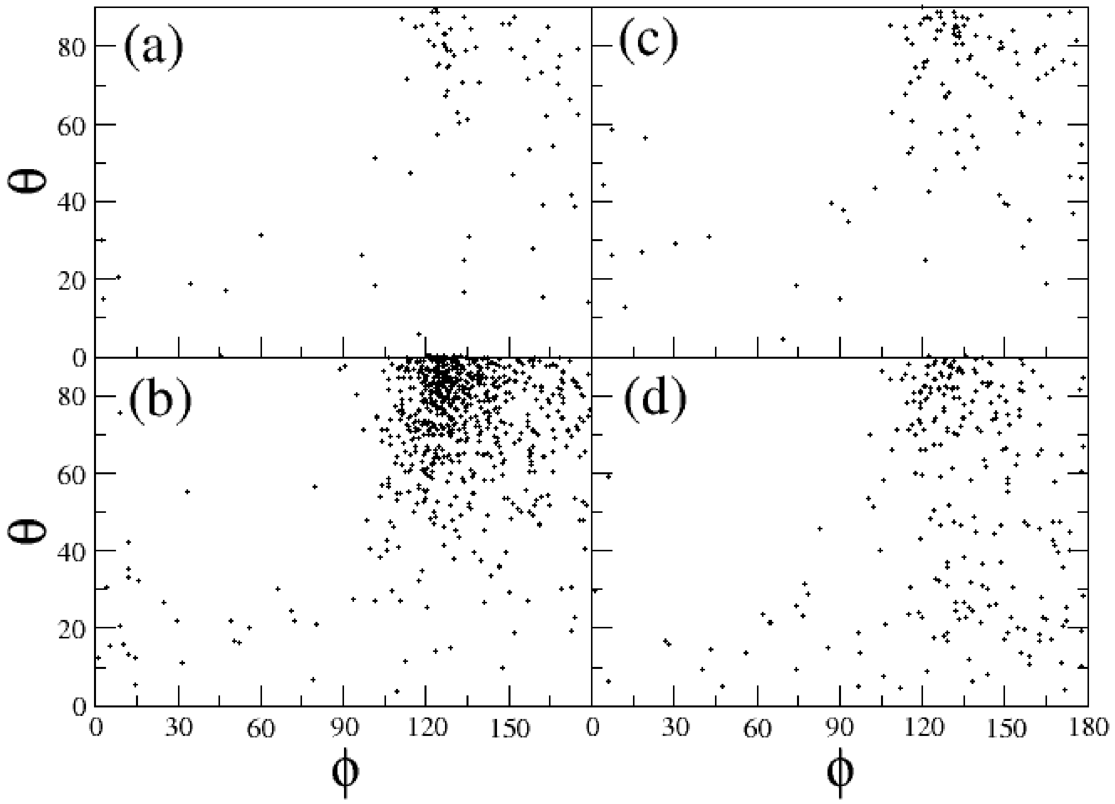

Figure S2. Relationships between the angles θ and ϕ (°) for amide oxygen atoms of Asn and Gln side-chains (**a**), for backbone carbonyl oxygen atoms (**b**), for carboxylate oxygen atoms of Asp and Glu side-chains (**c**), and for hydroxyl oxygen atoms of Ser, Thr and Tyr side-chains (**d**).

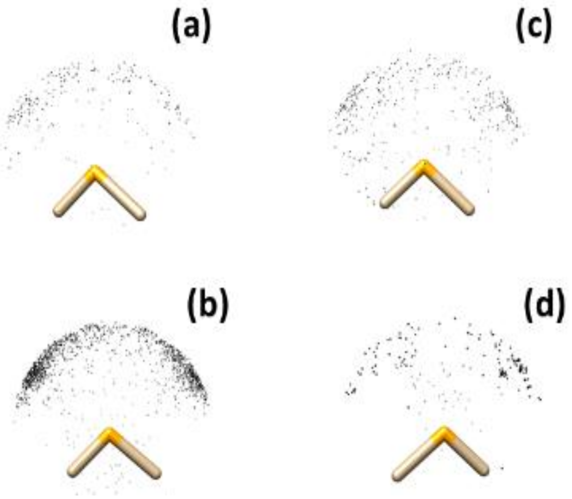

Figure S3. Superposition of all the oxygen atoms close to the selenium atom for amide oxygen atoms of Asn and Gln side-chains (**a**), for backbone carbonyl oxygen atoms (**b**), for carboxylate oxygen atoms of Asp and Glu side-chains (**c**), and for hydroxyl oxygen atoms of Ser, Thr and Tyr side-chains (**d**).
